# Chrysin: A Comprehensive Review of Its Pharmacological Properties and Therapeutic Potential

**DOI:** 10.3390/ph18081162

**Published:** 2025-08-05

**Authors:** Magdalena Kurkiewicz, Aleksandra Moździerz, Anna Rzepecka-Stojko, Jerzy Stojko

**Affiliations:** 1 Department of Toxicology, Toxicological Analysis and Bioanalysis, Faculty of Pharmaceutical Sciences in Sosnowiec, Medical University of Silesia in Katowice, 41-200 Sosnowiec, Poland; amozdzierz@sum.edu.pl (A.M.); jstojko@sum.edu.pl (J.S.); 2 Faculty of Medicine, Branch in Bielsko-Biała Medical University of Silesia in Katowice, 43-382 Bielsko-Biała, Poland; 3 Department of Drug and Cosmetics Technology, Faculty of Pharmaceutical Sciences in Sosnowiec, Medical University of Silesia in Katowice, 41-200 Sosnowiec, Poland; annastojko@sum.edu.pl

**Keywords:** flavonoids, chrysin, effects of chrysin, absorption of phytochemicals

## Abstract

Flavonoids constitute a broad class of naturally occurring chemical compounds classified as polyphenols, widely present in various plants, fruits, and vegetables. They share a common flavone backbone, composed of two aromatic rings (A and B) connected by a three-carbon bridge forming a heterocyclic ring (C). One representative flavonoid is chrysin, a compound found in honey, propolis, and passionflower (*Passiflora* spp.). Chrysin exhibits a range of biological activities, including antioxidant, anti-inflammatory, anticancer, neuroprotective, and anxiolytic effects. Its biological activity is primarily attributed to the presence of hydroxyl groups, which facilitate the neutralization of free radicals and the modulation of intracellular signaling pathways. Cellular uptake of chrysin and other flavonoids occurs mainly through passive diffusion; however, certain forms may be transported via specific membrane-associated carrier proteins. Despite its therapeutic potential, chrysin’s bioavailability is significantly limited due to poor aqueous solubility and rapid metabolism in the gastrointestinal tract and liver, which reduces its systemic efficacy. Ongoing research aims to enhance chrysin’s bioavailability through the development of delivery systems such as lipid-based carriers and nanoparticles.

## 1. Introduction

Flavonoids are naturally occurring chemical compounds widely distributed in the plant kingdom, recognized for their beneficial health properties. Among them, chrysin holds a prominent position as a flavonoid with strong biological potential, demonstrating antioxidant, anti-inflammatory, and anticancer activities. Owing to its chemical structure, chrysin is capable of modulating various cellular processes; however, its efficacy is largely dependent on its ability to penetrate cellular membranes. Understanding the mechanisms underlying its biological activity and transport is crucial for the development of novel therapeutic strategies aimed at supporting the treatment of chronic and lifestyle-related diseases.

## 2. Flavonoids

Flavonoids represent a vast group of phenolic secondary metabolites. They are synthesized in spore-bearing and vascular plants, as well as in mosses and algae [1]. A significant amount of flavonoids is also found in beverages such as tea (particularly green tea) and red wine [2]. These compounds are typically characterized by distinct coloration—depending on the specific flavonoid—which contributes to the pigmentation of the fruits in which they occur. In plants, flavonoids function as growth regulators and phytohormones, optimize photosynthetic processes [3,4], and serve protective roles by shielding plant cells from solar radiation and the harmful effects of fungi and insects [5,6,7]. Certain flavonoids, including nobiletin and chrysin, have been shown to modulate peroxisome proliferator-activated receptors (PPARγ and PPARα), leading to the suppression of inflammation, activation of apoptotic pathways, and enhancement of lipid metabolism. These interactions play a regulatory role in key biological pathways involved in angiogenesis and the immune response [8,9].

## 3. Chrysin

### 3.1. Chemical Structure

**Figure 1:**
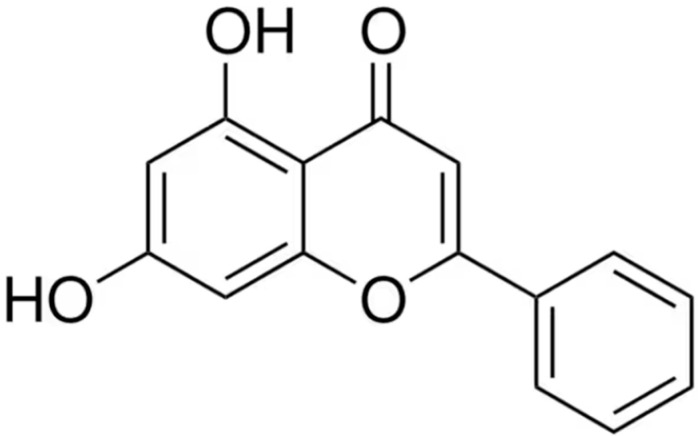
The chemical structure of chrysin (5,7-dihydroxyflavone).

Chrysin, also known as 5,7-dihydroxyflavone, is a member of the flavonoid family. It is a naturally occurring compound extracted from various plants, including *Passiflora caerulea (*blue passionflower*)*, *Passiflora incarnata* (purple passionflower), *Oroxylum indicum*, *Cytisus multiflorus*, *Crataegus oxyacantha*, *Pelargonium crispum*, *Scutellaria immaculata*, and *Alpinia oxyphylla* [10,11]. Additionally, chrysin is found in different types of honey and propolis [12], as well as in certain edible mushrooms such as *Pleurotus ostreatus* (oyster mushroom) [13]. Chemically, chrysin belongs to the class of dihydroxyflavones, characterized by hydroxyl groups attached solely to the aromatic A-ring—specifically at the 5 and 7 positions—distinguishing it from other flavones (Figure 1). In plants, the biosynthesis of chrysin originates from the amino acid phenylalanine, which is converted into cinnamic acid through the action of the enzyme L-phenylalanine ammonia-lyase (PAL) [14,15]. Chrysin is a lipophilic compound with a low molecular weight (MW = 254.24 g/mol). Its aqueous solubility is limited, with values of 0.06 ± 0.1 mg/mL at pH 6.5 and 0.058 ± 0.04 mg/mL at pH 7.4. In the human body, chrysin exhibits poor absorption, is rapidly metabolized, and promptly eliminated, resulting in very low bioavailability [16]. This is primarily due to its high susceptibility to phase II metabolic enzymes, particularly UDP-glucuronosyltransferases (UGTs), which catalyze glucuronidation and sulfonation reactions leading to its rapid clearance [17,18,19].

**Table 1:** Comparison of main flavonoids: main sources, in vitro efficacy, bioavailability, key therapeutic benefits, and limitations.

Characteristic	Chrysin	Quercetin	Apigenin	Catechins (e.g., EGCG from Green Tea)	Genistein
Main sources	Propolis, honey, passionflower (Passiflora) [20,21]	Onion, apples, broccoli, capers, grapes [20,21]	Chamomile, celery, parsley, oranges [20,21].	Green tea, black tea, cocoa, berries [20,21].	Soy and its products (tofu, soy milk) [20,21].
In vitro efficacy	High anti-inflammatory, antioxidant, and anticancer activity. Aromatase inhibitor [20,21].	Very high antioxidant and anti-inflammatory activity. Antiviral and anticancer effects [20,21].	Strong anxiolytic, calming, anti-inflammatory, and anticancer effects [20,21].	Exceptionally strong antioxidant and anticancer effects. Supports metabolism and cardiovascular health [20,21].	Estrogen-like activity (phytoestrogen). Strong anticancer properties (breast and prostate cancer) [20,21].
Bioavailability	Very low. It is rapidly metabolized in the intestines and liver (first-pass effect), which drastically limits its concentration in the bloodstream. Delivery systems (e.g., nanoparticles) are required [22].	Low to moderate. Better than chrysin, but it also undergoes extensive metabolism. Its absorption improves in the presence of fats and vitamin C [23,24].	Low. Similar metabolism issues as with other flavonoids, but it shows the ability to cross the blood-brain barrier [25,26].	Moderate. Bioavailability is variable and relatively low, but sufficient to produce biological effects with regular tea consumption [27,28].	Moderate to high. One of the most well-absorbed flavonoids, especially in Asian populations that regularly consume soy [29,30].
Key therapeutic benefits	Potential anticancer effects (mainly observed in laboratory studies).	Support for the cardiovascular system (blood pressure reduction).	Natural calming and sleep aid (acts on GABA receptors).	Strong cancer prevention.	Relief of menopause symptoms.
	Neuroprotective and anxiolytic properties.	Reduction of inflammation and allergy symptoms.	Cancer prevention.	Heart and brain protection (neuroprotective and cardioprotective effects).	Osteoporosis prevention.
	Support in bodybuilding as an aromatase inhibitor (effect not clinically confirmed) [21].	Immune support (antiviral activity) [20,21,22,23].	Skin health (used in cosmetics) [25,31].	Support for weight management and type 2 diabetes treatment [27,32].	Significant role in prevention and treatment of hormone-dependent cancers [20,21,29].
Limitations and considerations	The main limitation is extremely low bioavailability, which undermines its therapeutic effectiveness in vivo without advanced delivery systems [22].	It may interact with certain medications (e.g., blood thinners) and chemotherapy [23,24].	It may enhance the effects of sedative medications [25,26].	High doses may be toxic to the liver. It may also inhibit iron absorption [33].	Due to its phytoestrogenic effects, its supplementation is controversial in patients with hormone-dependent cancers [21,29,30].

Chrysin is a flavonoid with very interesting biological potential; however, its practical application is severely limited by pharmacokinetic barriers, primarily its very poor bioavailability and rapid metabolism. In comparison, flavonoids such as quercetin, EGCG, and genistein—although not without their own limitations—exhibit better bioavailability, which translates into more well-documented and credible therapeutic benefits in human studies. Apigenin, on the other hand, stands out due to its unique effects on the nervous system [34]. Comparative properties are listed in Table 1.

### 3.2. Chemical Properties Derived from the Structure

The antioxidant activity of chrysin is primarily associated with the presence of a double bond between the carbon atoms at positions 2 and 3 (C2=C3), as well as a carbonyl group (C=O) at the C4 carbon atom [35,36]. The absence of –OH groups at positions C3 and C4 in the B ring of the chrysin molecule contributes to many of its properties—ranging from anti-inflammatory to antitoxic effects [37,38]. The –OH groups located at the C5 and C7 positions are mainly responsible for chrysin’s ability to scavenge reactive oxygen species (ROS). These groups, especially the –OH group at position 7, can donate hydrogen atoms, thereby neutralizing free radicals and reactive oxygen species. However, the lack of a hydroxyl group in the B ring significantly limits its antioxidant potential compared to other flavonoids, such as quercetin or luteolin [35,36]. The biological activity of chrysin can be enhanced through various modifications—for instance, introducing hydrophobic chains at the C5 and C7 positions significantly improves its anti-inflammatory activity [39,40,41].

These modifications include, among others:

- Hydroxylation: The introduction of additional hydroxyl groups, especially into the B ring, leads to the formation of other well-known flavonoids, such as apigenin (–OH group at the 4′ position) or luteolin (–OH groups at the 3′ and 4′ positions). This process can mimic natural biosynthetic pathways found in plants [42].
- Prenylation: The attachment of prenyl groups to the chrysin backbone significantly increases its lipophilicity and its ability to penetrate cell membranes, which often results in enhanced anticancer and anti-inflammatory activity [42,43].
- Glycosylation: The attachment of sugar molecules to hydroxyl groups improves the compound’s water solubility and affects its bioavailability [44].
- Synthesis of metal complexes: The hydroxyl and carbonyl groups in chrysin can chelate metal ions, forming complexes with new, unique properties, including antimicrobial and antioxidant activities [43,45].

The use of the chrysin molecular scaffold with attached nitric oxide-donating prodrugs enhanced its vasoprotective effects and promoted angiogenesis. Methylation at the C5 and C7 positions resulted in higher therapeutic efficacy of a chrysin analog in the treatment of acute lymphoblastic leukemia [46]. Meanwhile, butyl, octyl, propyl, and tolyl derivatives of the –OH groups at C5 and C7 were responsible for the compound’s antiglycemic effect [47]. Chrysin derivatives containing fluorine atoms, on the other hand, demonstrated more effective antimicrobial [48] and anticancer activities [49,50,51].

### 3.3. Chrysin as a Precursor for the Synthesis of Prodrugs and Carriers

The use of chrysin as a precursor in prodrug synthesis allows for improved pharmacokinetics and reduced side effects:

Ester and amide prodrugs: The synthesis of chrysin esters and amides with various functional groups increases metabolic stability and bioavailability, as confirmed by preclinical studies in animal models of metabolic and cancer-related diseases [52].

Nanoformulations and carriers: Chrysin is often incorporated into nanoscale delivery systems (niosomes, lipid nanoparticles, nanovesicles), which enhance its solubility and efficacy in the treatment of cancers, neurodegenerative diseases, and infections [53].

Chrysin is also used as a starting substrate for the synthesis of flavonoid derivatives with anticancer, antibacterial, or neuroprotective activity:

Derivatization in the B ring: An example is the synthesis of 3′,4′-dihydroxychrysin derivatives, which exhibit stronger antioxidant and anticancer effects [54].

Metal chelation: Chrysin forms complexes with metal ions (Cu, Fe, Zn) that exhibit enhanced antioxidant and antimicrobial activities, opening up possibilities for the synthesis of metalloflavonoid-based drugs [55].

Explanation of the Mechanism

The diagram illustrates the key process by which chrysin protects cells from oxidative damage. This mechanism proceeds in three main steps:

1. Hydrogen atom donation: The chrysin molecule encounters a highly reactive free radical (in the diagram: a hydroxyl radical, •OH). The hydroxyl group at the C7 position of chrysin, which is crucial for this activity, donates its hydrogen atom to the free radical.

2. Radical neutralization: The free radical, upon receiving the hydrogen atom, is converted into a stable and harmless water molecule (H_2_O). Thus, the threat to the cell is neutralized.

3. Resonance stabilization: After donating the hydrogen atom, chrysin itself becomes a radical. However, it is very stable and poorly reactive because the unpaired electron is dispersed (delocalized) throughout the entire ring structure, including the A-ring and the carbonyl group. This process, called resonance stabilization, prevents the further propagation of damage.

**Figure 2:**
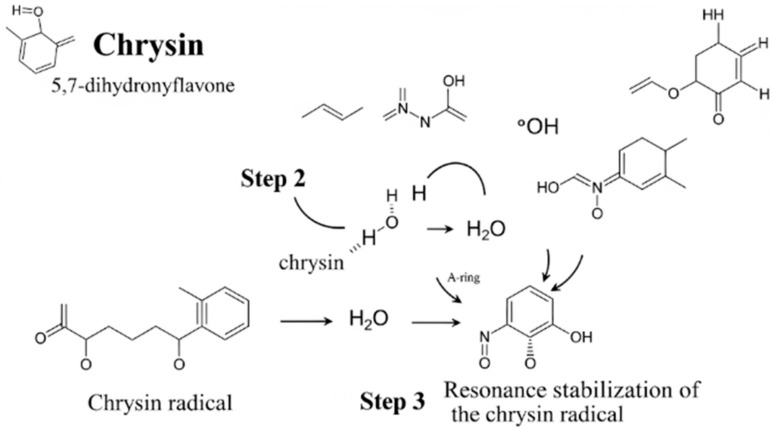
A simplified mechanism of free radical (ROS) scavenging by chrysin.

Through this mechanism, chrysin effectively terminates the chain reaction of damage to important cellular components, such as proteins, lipids, and DNA [56]. Simplified mechanism of free radical scavenging by chrysin can be found in Figure 2.

## 4. Mechanisms of Action of Chrysin

Chrysin possesses very strong anti-asthmatic properties because it inhibits inducible nitric oxide synthase (iNOS) and the nuclear factor NF-κB, which plays a significant role in immune and inflammatory processes such as bronchial asthma [57,58].

The anti-allergic effects of chrysin have been observed in studies on atopic dermatitis. Chrysin suppresses the progression of this condition by regulating the activity of mitogen-activated protein kinase p38, NF-κB [59], STAT1, and IL-33, as well as by inhibiting histamine release through the regulation of calcium ion concentration and the production of pro-inflammatory cytokines [60,61].

Osteoporosis is a disease primarily affecting older adults and typically requires treatment based on hormone replacement therapy. Unfortunately, this often comes with many side effects, so alternative therapeutic options are currently being sought. Chrysin shows promising potential in this regard [62].

Studies conducted on a rat model with induced osteoporosis suggest that this flavonoid may be a promising alternative for treating bone loss and its associated effects. In this study, chrysin increased the diameter of the basic bone-building units, such as the trabeculae of the femoral epiphyses, and also reduced the cross-sectional area of the bone marrow cavity as well as the overall ratio of this area to the total cross-sectional area of the bone shaft [63]. Protective effects of chrysin were also observed in measurements of calcium and phosphorus concentrations in bone tissue, which are important in osteoblastic processes [64]. Additionally, chrysin influences the differentiation of bone cells by activating the mitogen-activated protein kinase signaling pathway (ERK/MAPK) [11,65].

Chrysin exerts cardioprotective effects by modulating certain cellular signaling pathways involved in inflammation, oxidative stress [66], and dysfunction of vascular cells [67]. In individuals suffering from cardiovascular diseases, inflammatory markers are significantly elevated [68,69].

Chrysin demonstrates anti-inflammatory action through several mechanisms: it suppresses cyclooxygenase-2 (COX-2), an enzyme involved in prostaglandin synthesis that promotes inflammation [70]; it inhibits phosphorylation and degradation of IκB-α, as well as the translocation of NF-κB, and reduces levels of TNF-α and IL-1β by inhibiting NF-κB expression [71,72].

Oxidative stress undoubtedly contributes to the development of cardiovascular diseases such as atherosclerosis, hypertension, and cardiomyopathies. Reactive oxygen species (ROS) are responsible for hyperlipidemia and atherosclerosis, ultimately leading to thrombosis [73]. Chrysin protects against ROS by reducing lipid peroxidation levels in the liver and increasing both enzymatic and non-enzymatic antioxidant levels [74,75]. It also increases the expression of NOX protein while decreasing the activity of NF-κB and the production of nitric oxide (NO) [76].

Endothelial cells lining blood vessels are responsible for proper fluid flow, vascular tone, and overall cardiovascular homeostasis. Their dysfunction initiates various cardiovascular diseases [77]. Recent studies show that chrysin supports vascular relaxation by activating the cyclic guanosine monophosphate (cGMP) pathway—this pathway influences vascular tone, causing vessel relaxation and thereby improving transport functions [78,79].

Chrysin also exhibits neuroprotective effects in neurological disorders such as epilepsy, neuronal apoptosis, neuroinflammation [80], anxiety [81], depression [82], multiple sclerosis [83], Parkinson’s disease, Alzheimer’s disease, cognitive deficits, and various other age-related neurological conditions [84]. In epilepsy models using rats with induced seizures, chrysin counteracted oxidative stress, reduced neuronal apoptosis, and increased expression of nuclear factor erythroid 2-related factor 2 (Nrf2) and heme oxygenase-1 (HO-1) [80].

Chrysin can inhibit neuronal apoptosis by downregulating the expression of key proteins involved in programmed cell death, including Bax, cytochrome C, caspase-3, caspase-8, and p53 at the gene transcription level [85,86,87]. Since these molecules play critical roles in apoptosis, their inhibition prolongs healthy neuronal activity [88].

Studies on a mouse model of Parkinson’s disease showed that chrysin provides neuroprotection against MPTP-induced loss of dopaminergic neurons. It also alleviates damage caused by oxidative stress, neuroinflammation, and impaired Na^+^/K^+^-ATPase activity [89,90].

Neuroinflammation and synaptic plasticity lead to oxidative damage and the release of pro-inflammatory cytokines, especially TNF-α and IL-1 (α, β) [91]. Treatment with chrysin in rats modulated inflammatory and immune mediators such as TNF-α, IFN-γ, IL-1β, and IL-6 [92]. Chrysin suppressed neuroinflammation in activated microglia by modulating levels of NO, iNOS, COX-2, TNF-α, IL-1β, and NF-κB [93].

Positive effects of chrysin on neuroinflammation were demonstrated in primary cultures of embryonic mouse cortical neurons, the BV2 microglial cell line, and primary mouse microglia. Chrysin inhibited NO and TNF-α production; these effects were linked to reduced levels of C/EBPδ protein, mRNA expression, and DNA-binding activity, without affecting nuclear levels of C/EBPβ and p65 or their DNA-binding activity, suggesting C/EBPδ as a potential mediator of chrysin’s effects [94].

Chrysin also has applications in the treatment of anxiety. Studies conducted on menopausal rats showed that chrysin exhibits anxiolytic effects by acting on GABA receptors [95,96].

Depression is another mental health disorder that responds positively to chrysin treatment in animal models. In rat models, chrysin was shown to lower serum corticosterone and malondialdehyde (MDA) levels while increasing glutathione (GSH), superoxide dismutase (SOD), glutathione peroxidase (Gpx), glutathione reductase (GR), and catalase (CAT). In a mouse model of chronic depression, chrysin also demonstrated antidepressant effects by reducing levels of TNF-alpha, IL-1β, IL-6, and kynurenine, as well as lowering levels of corticotropin-releasing hormone and adrenocorticotropic hormone in plasma, particularly in the prefrontal cortex and hippocampus. Moreover, chrysin increased serotonin (5-HT) levels and reduced the activity of indoleamine 2,3-dioxygenase. It also decreased the expression of caspases-3 and -9 [97,98].

Experimental use of chrysin in multiple sclerosis yielded positive results as well. In an animal model of autoimmune encephalomyelitis, which mimics the pathogenesis of multiple sclerosis in humans, treatment with chrysin reduced histone deacetylase activity, levels of glycogen synthase kinase 3 beta (GSK-3β), IFN-γ, IL-17, TNF, and histone acetyltransferases HAT3 and HAT4—factors important in the analysis of neurological disease pathogenesis and neurogenesis [99].

Endometriosis is a common chronic disease primarily affecting women of reproductive age. It is characterized by abnormal growth and proliferation of endometrial cells outside the uterine cavity [100]. Although endometriosis is not classified as a cancer, it exhibits certain features such as proliferation, angiogenesis, migration, and invasion, which have molecular similarities to those observed in cancer cells, as well as the formation of inflammation and oxidative stress. This has led to the hypothesis that the disease can be alleviated by compounds with documented anti-inflammatory and anticancer activities, such as flavonoids [101].

Experiments conducted on human endometrial cells, female mice, and rats involving flavonoids such as chrysin [102], apigenin [103], naringenin [104], and myricetin [105] showed that these compounds can target molecular pathways related to oxidative stress, strengthening them and predisposing proliferating cells outside the uterus to enter apoptotic pathways. Additionally, other studied flavonoids—xanthohumol [106], isoliquiritigenin, and luteolin—beneficially affect angiogenesis in endometrial cells by inhibiting blood vessel formation through mechanisms involving VEGF. The anti-inflammatory effects of flavonoids on the endometrium were confirmed with apigenin, isoliquiritigenin, and luteolin, which act via NF-κB, TNF-α, IL-1, IL-6, and macrophages associated with endometriosis, thereby inhibiting the progression of the disease [107,108].

By targeting receptors such as PPAR, AhR, and NR4A1, flavonoids demonstrate the ability to modulate both metabolic and inflammatory pathways, offering a multifaceted approach to treating endometriosis. Flavonoids can selectively interact with pathophysiological molecules and pathways involved in this disease. Therefore, utilizing the therapeutic properties of flavonoids may lead to new strategies for endometriosis treatment [109,110,111].

### Anticancer Activity in Various Types of Cancer

The anticancer activity of chrysin warrants a separate discussion due to its particular significance and well-documented therapeutic potential. Numerous studies have shown that chrysin affects key mechanisms of carcinogenesis—such as inhibiting cancer cell proliferation, inducing apoptosis, and enhancing the effects of cytotoxic drugs. The diversity of these effects, along with the growing interest in chrysin as a potential anticancer agent, justifies presenting this topic in a dedicated subsection.

The anticancer activity of chrysin is primarily based on the activation of pathways involved in programmed cell death. The pro-apoptotic effects of chrysin have been documented in a wide range of cancers, including cervical cancer, leukemia, esophageal cancer, breast cancer, lung cancer, and prostate cancer [112]. At the molecular level, its anticancer effects mainly involve activation of caspases through binding with tumor necrosis factor-related apoptosis-inducing ligand (TRAIL) [113], as well as enhancing TRAIL-induced activation of pro-apoptotic caspases and inhibiting the PI3K/Akt signaling pathway [114].

Prostate cancer is one of the most common cancers among men. Chrysin’s action is based on inducing apoptosis through mitochondrial damage—raising reactive oxygen species (ROS) levels, which disrupt mitochondrial membrane integrity, leading to cytochrome c release and triggering programmed cell death. Additionally, it causes cell cycle arrest in cancer cells, reduces expression of MAPK and PI3K/Akt signaling pathways, and disrupts overall cell proliferation [115,116].

Stomach cancer is the third leading cause of cancer-related deaths worldwide. Various factors contribute to the progression of this cancer, and the enzyme TET1 is one of them. These enzymes are involved in oxidizing 5-methylcytosine to 5-hydroxymethylcytosine and participate in epigenetic modification. In a study conducted on MKN45 cell lines, chrysin promoted TET1 and 5-hydroxymethylcytosine expression, which stimulated apoptosis and disrupted the migration of gastric cancer cells [117].

Chrysin’s effects in lung cancer include decreasing the expression of TLR4 and Myd88 in the signaling cascade from activated receptor to the cell interior. TLR4 is part of the Toll-like receptor family, membrane proteins that serve as the first line of defense against pathogens, while Myd88 is the first protein in the intracellular signaling pathway. Chrysin suppresses inflammation by reducing NF-κB expression and pro-inflammatory factors such as IL-1β, IL-6, TNF-α, and IL-10; it also inhibits cancer cell survival and metastasis capabilities [118,119].

In the case of cervical cancer, chrysin also confirms its anticancer effects. This activity was studied in HeLa cells. Its mechanism of action is based on increasing the level of the p53 protein in HeLa cells, along with enhanced interaction with the p21 protein, which blocks the cell cycle. Chrysin induces cell cycle arrest at the G2/M phase. Increased expression of the tumor suppressor p16 in cells treated with lower concentrations of chrysin causes senescence, which is a crucial event in cancer prevention [120,121].

Breast cancer is one of the most common malignant tumors in women and is the leading cause of death among women aged 20–50 years [122]. Current research indicates a strong association with the human epidermal growth factor receptor 2 (HER2), which has tyrosine kinase activity. HER2 amplification in breast cancer defines the HER2-positive breast cancer subtype. Although HER2-positive breast cancer accounts for only about 20% of all breast cancer cases, it remains the most incurable subtype in clinical practice. In an experiment aimed at testing the anticancer effects of pyrotinib combined with chrysin, it was confirmed that adding chrysin positively enhanced the inhibition of HER2-positive breast cancer growth both in vitro and in vivo, compared to pyrotinib alone [123].

Hepatocellular carcinoma: Studies of anticancer mechanisms in hepatocellular carcinoma showed that the biological activity exerted by chrysin was mainly attributed to its effect on hexokinase (HK-2). As HK-2 levels decreased, chrysin inhibited glycolysis (which impairs glucose uptake and lactate production) in the tumor and activated mitochondria-related apoptosis. It induces apoptosis by disrupting the interaction between hexokinase 2 and the VDAC1-1 protein, impairing mitochondrial function and releasing cytochrome c into the cytoplasm. Considering that HK-2 overexpression is observed in most hepatocellular carcinoma tissues, these results suggest that chrysin or its analogs could be effective drugs in treating hepatocellular carcinoma [124].

Melanoma is one of the cancers whose incidence and mortality rates have significantly increased in recent decades. A study investigating the effects of chrysin on human A375 melanoma cells showed that chrysin induces both apoptosis and autophagy by modulating the mTOR/S6K pathway. This effect is also associated with cell cycle arrest at the G2/M phase, leading to the inhibition of tumor cell proliferation. Based on these observations, the authors suggest that chrysin has therapeutic potential as an adjuvant treatment for melanoma by regulating key signaling pathways responsible for tumor cell survival and death [125].

Colorectal cancer is a heterogeneous disease whose incidence has risen in recent years. Both molecular and pathological characteristics determine the prognosis and response of colorectal cancer cells to therapy. Until now, 5-fluorouracil (5-FU) has been commonly used in colorectal cancer treatment; however, resistance to its effects and adverse side effects have limited its use [126]. A study using chrysin as an alternative to 5-fluorouracil demonstrated that chrysin can stimulate autophagy in colorectal cancer cells. Autophagy, a process of cellular “self-digestion,” plays a critical role in cancer treatment. In this study, chrysin was shown to promote the generation of reactive oxygen species (ROS) and reduce mTOR expression, thereby stimulating autophagy [127].

Bladder cancer ranks second among the most common cancers in developed countries [128]. Chemotherapy is not recommended for this cancer due to chemoresistance and side effects, making research into new strategies, such as the use of phytochemicals, a promising avenue. Inducing endoplasmic reticulum (ER) stress through activation of the unfolded protein response (UPR)—a multifunctional PERK-dependent signaling pathway—stimulates the intrinsic apoptosis pathway by regulating increased caspase-3 and caspase-9 activity and inhibits the STAT3 signaling pathway. Anti-apoptotic factors such as Bcl-2, Mcl-1, and Bcl-xl are downregulated by chrysin in bladder cancer cells. Notably, chrysin significantly reduces cell viability by inducing ER stress through stimulation of UPR, PERK, ATF4, and eIF2α [129].

The following table (Table 2) summarizes the key biological activities of chrysin documented in the scientific literature, providing readers with a quick overview of its therapeutic potential.

## 5. Methods of Phytochemical Transport into the Cell

Cancer therapies remain an unfinished and continuously evolving topic. In their design, a well-established mechanism of action for new compounds tested in vitro and in vivo is undoubtedly crucial, but so is the method of delivering these compounds to the body. For phytochemicals such as chrysin, several methods of delivering the substance to cells have already been developed: micelles, dendrimers, polymer nanoparticles, and solid lipid nanoparticles. Micelles are amphiphilic copolymers ranging in size from 10 to 100 nm. They are characterized by efficient cellular uptake [130]. In an experiment examining the effectiveness of chemotherapy using micelles loaded with chrysin combined with the chemotherapeutic agent docetaxel, it was observed that these micelles, together with the drug, effectively inhibit the migration and invasion of cancer stem cells. The use of such nanoparticles was tested on cells from a specific cancer—breast cancer—with positive results [131]. An additional advantage—besides promoting cytotoxicity against cancer cells—is their size, which prevents uptake by macrophages and filtration by the glomeruli, allowing effective delivery of the anticancer drug to the target cells [132].

Another biomaterial experimentally used to deliver potential drugs to cancer cells is polyurea dendrimers. These are three-dimensional polymers with urea segments in the polymer backbone and amino groups located peripherally. They exhibit several favorable properties for interactions with the human body (biocompatibility, biodegradability), are pH-sensitive, and are highly water-soluble. Their application in delivering chrysin was studied in ovarian cancer, where chrysin encapsulated in dendrimers induced oxidative stress and decreased the viability of ovarian cancer cells [133].

Additionally, similar to micelles, they do not exhibit toxic effects on healthy cells, which increases their “attractiveness” as a method for drug delivery to cells [134]. Another method of introducing drugs into cells is the use of polymer nanoparticles. These structures have a core–shell architecture that degrades in an aqueous environment. The hydrophilic part provides stability to the nanoparticle, while the hydrophobic core surrounds the anticancer drug. Polymers that have already found medical applications include primarily poly(ε-caprolactone) (PCL), polyglycolide (PGA), and polylactides (PLA) [135]. Polymer nanoparticles containing chrysin were studied for delivering it to breast cancer cells. A major advantage of these nanoparticles is their high cellular uptake. Nanoparticles also serve as a platform that can “carry” more than one anticancer drug [136]. When investigating the anticancer effects of chrysin and curcumin on colorectal cancer cells, PLGA-PEG nanoparticles loaded with these two compounds were used. The experiment demonstrated that the phytochemicals encapsulated in the nanoparticles decreased the expression of hTERT, inhibiting colorectal cancer cell growth, and also suppressed metastasis by reducing the expression of MMP-2 and MMP-9 [137]. Nanoparticles containing chrysin and curcumin also enhance the expression of tissue inhibitors of metalloproteinases (TIMP), which regulate MMP activity [138]. The characteristics of chrysin delivery systems are presented in Table 3.

## 6. The Most Important Limitations in the Use of Chrysin

### 6.1. Extremely Low Bioavailability and Poor Absorption

This is a fundamental and critical problem. After oral administration, chrysin is very poorly absorbed from the gastrointestinal tract due to:

Low water solubility: Chrysin dissolves poorly in bodily fluids, which is the first essential step for effective absorption.

Rapid first-pass metabolism: The small amount of chrysin that manages to be absorbed is immediately and extensively metabolized in the intestines and liver. This leads to the formation of inactive or much less active metabolites (glucuronides and sulfates) before the compound reaches the bloodstream [146,147].

As a result, the concentration of active, free chrysin in the blood is negligible and often insufficient to induce the desired therapeutic effects observed in in vitro studies [148].

### 6.2. Lack of Solid Evidence from Clinical Trials in Humans

Most impressive results regarding chrysin’s anticancer, anti-inflammatory, or neuroprotective properties come from cell line studies (in vitro) or animal models (in vivo). These studies often use high concentrations or special administration routes (e.g., injections) that bypass the bioavailability issue. However, high-quality randomized clinical trials confirming these benefits in humans after standard oral supplementation are lacking.

### 6.3. Questionable Efficacy as an Aromatase Inhibitor in Humans

Chrysin is popular among athletes and bodybuilders as a purported natural aromatase inhibitor, meant to block the conversion of testosterone to estrogen [149]. However, clinical studies in humans have not confirmed this efficacy. Oral intake of chrysin was shown not to significantly affect estrogen or testosterone levels, which directly results from its minimal bioavailability [150].

### 6.4. Potential Drug Interactions

Being metabolized by the same enzymes as many drugs (cytochrome P450), chrysin theoretically could interact by affecting their concentration and action. Although this risk is low with standard supplementation (due to poor absorption), it may become relevant when advanced delivery systems are used that increase blood concentrations [151].

### 6.5. Lack of Regulation and Standardization of Supplements

Chrysin dietary supplements are not subject to the strict controls applied to drugs. This means that dosage, purity, and product quality can vary significantly on the market, and the actual content of the active substance may differ from what is declared [152].

In summary, despite the promising biological potential of chrysin, its practical application is currently severely limited by its inherent pharmacokinetic properties. Until effective and safe delivery systems (such as nanoparticles or liposomes) are developed and widely implemented to overcome the issue of low bioavailability, the benefits of chrysin supplementation remain largely unproven and doubtful.

To fully appreciate the therapeutic potential of chrysin, it is valuable to broaden the perspective by considering other classes of natural compounds that, despite differing chemical structures, exhibit a remarkably similar biological activity profile. A prime example of such compounds is isocoumarins—a group of naturally occurring substances whose pharmacological properties have become the focus of intense research.

#### 6.5.1. Anti-Inflammatory and Antioxidant Activity

Similar to chrysin and other flavonoids, isocoumarins exhibit strong anti-inflammatory effects. Their mechanism of action is largely based on the suppression of the transcription factor NF-κB, a major regulator of the cellular inflammatory response. They also inhibit the production of key pro-inflammatory mediators such as TNF-α, IL-6, and cyclooxygenase-2 (COX-2), which parallels the mechanisms described for chrysin [153].

The antioxidant potential of isocoumarins, which is critical in combating various pathological conditions, is attributed to the presence of phenolic groups in their structure. These groups enable effective neutralization of reactive oxygen species (ROS). Through a hydrogen atom donation mechanism, isocoumarins interrupt the oxidative stress cascade, thereby protecting cells from DNA, lipid, and protein damage—an essential property they share with flavonoids [154].

#### 6.5.2. Neuroprotective Potential

Of particular promise are recent reports highlighting the neuroprotective effects of isocoumarins. Studies indicate that these compounds may protect neuronal cells through multiple mechanisms, including reducing the toxicity of β-amyloid deposits, preserving mitochondrial function, and inhibiting apoptotic pathways in neurons. This suggests their potential application in the treatment of neurodegenerative diseases such as Alzheimer’s and Parkinson’s disease, which aligns with the therapeutic promise observed for chrysin [155].

#### 6.5.3. Summary and Contextual Relevance

An analysis of isocoumarin properties—despite their classification as a distinct chemical group—reinforces the overarching conclusions of this review. Nature offers a vast repertoire of bioactive compounds capable of modulating key cellular pathways essential for human health. Their multi-targeted biological activity and typically low toxicity profile make them attractive candidates for further investigation. However, as with chrysin, many of these compounds face similar pharmacokinetic challenges, such as low bioavailability, underscoring the critical need for continued research into novel drug delivery systems.

## 7. Conclusions

The literature review clearly indicates that chrysin is a versatile bioactive compound with significant pharmacological potential. Its documented anticancer, anti-inflammatory, antioxidant, and neuroprotective properties make it a promising candidate for further research in the context of treating many lifestyle diseases. Crucial to its activity is the presence of hydroxyl groups at positions 5 and 7, which determine its antioxidant capabilities. At the same time, its low bioavailability and limited water solubility are major barriers to its clinical application. The analysis has also shown that the chemical structure of chrysin is an excellent starting point for the synthesis of new, more effective derivatives, which is a promising direction for the development of modern, sustainable medicinal chemistry.

### Perspectives and Future Research Directions

To fully exploit the therapeutic potential of chrysin, future research should focus on the following areas:

- Development of novel drug delivery systems: Research on formulations (e.g., nanoparticles, liposomes, cyclodextrin complexes) to increase the bioavailability and solubility of chrysin.
- Synthesis and biological evaluation of new derivatives: Design and synthesis of modified chrysin analogs with increased potency, selectivity towards biological targets (e.g., cancer cells), and improved pharmacokinetic properties.
- Studies on synergistic mechanisms: Evaluating the efficacy of chrysin in combination therapies with conventional chemotherapeutics to potentially reduce drug doses and limit their toxicity.
- Advanced preclinical and clinical trials: Conducting detailed studies in animal models and, in the longer term, well-designed clinical trials to confirm its efficacy and safety in humans.
- Exploration of new therapeutic targets: Investigating the effect of chrysin on other, less-studied signaling pathways and pathological processes, such as autophagy, cellular senescence, or metabolic disorders.

## Figures and Tables

**Table 2:** Summary of the main biological activities of chrysin.

Biological Activity	Main Mechanism of Action	Example Effects
Anticancer	Induction of apoptosis, inhibition of proliferation and angiogenesis, cell cycle arrest [112,115].	Inhibition of breast, prostate, lung, and colon cancer cell growth [112,116,122,126].
Antioxidant	Neutralization of free radicals (ROS/RNS) by donating a hydrogen atom from –OH groups [73,74,75].	Protection of cells against oxidative stress [73,74,75].
Anti-inflammatory	Inhibition of NF-κB and MAPK signaling pathways, reduction of pro-inflammatory cytokine production (e.g., TNF-α, IL-6) [70,71,72].	Reduction of inflammation in various disease models [70,71,72].
Neuroprotective	Protection of neurons from apoptosis, inhibition of neuroinflammation, antioxidant action in the CNS [91,92,94].	Potential application in neurodegenerative diseases (Alzheimer’s, Parkinson’s) [91,92,94].
Antiviral	Inhibition of viral replication, e.g., by inhibiting key viral enzymes [2,9].	Activity against influenza, HIV, Herpes Simplex viruses [2,9].
Anxiolytic	Modulation of GABA-A receptors (action similar to benzodiazepines) [95,96].	Calming and anti-anxiety effect without typical side effects [95,96].

**Table 3:** Overview of drug delivery systems for chrysin: effectiveness and key limitations.

Drug Delivery System	Efficacy	Limitations
Micelles	Improvement of chrysin solubility thanks to the hydrophobic core. Increased stability and bioavailability. Possibility of surface modification for targeted delivery [139,140,141].	Low drug encapsulation efficiency. Possibility of premature drug release due to micelle instability under certain biological conditions [139,140,141].
Dendrimers	Precise, controlled structure and size. High surface functionality enabling attachment of targeting molecules. Enhanced solubility and bioavailability of chrysin [142].	Potential toxicity, especially with higher-generation dendrimers and positive charges. Complex and costly synthesis process [142].
Polymeric nanoparticles	High encapsulation efficiency. Possibility of controlled, prolonged drug release. Protection of chrysin from degradation. Improvement of therapeutic efficacy in vivo [143,144].	Possibility of drug leakage during storage. Complexity of the manufacturing process. Potential issues with biodegradability and toxicity of certain polymers [143,144].
Solid Lipid Nanoparticles (SLNs)	High biocompatibility and low toxicity due to the use of physiological lipids. Feasibility of large-scale production. Protection of the drug from chemical degradation. Improved bioavailability following oral administration [145].	Lower encapsulation efficiency compared to polymeric nanoparticles. Tendency for drug expulsion during storage due to the crystalline structure of lipids [145].

## Data Availability

Data sharing is not applicable.
